# Supplementary figures and images for: Nutrition-related knowledge, attitudes, practice, and mental health status of people living with HIV in Dubai, United Arab Emirates: A cross-sectional descriptive study
Source: PLoS One. 2026 Mar 4;21(3):e0344288. doi: 10.1371/journal.pone.0344288 (PMC12959681; doi:10.1371/journal.pone.0344288)

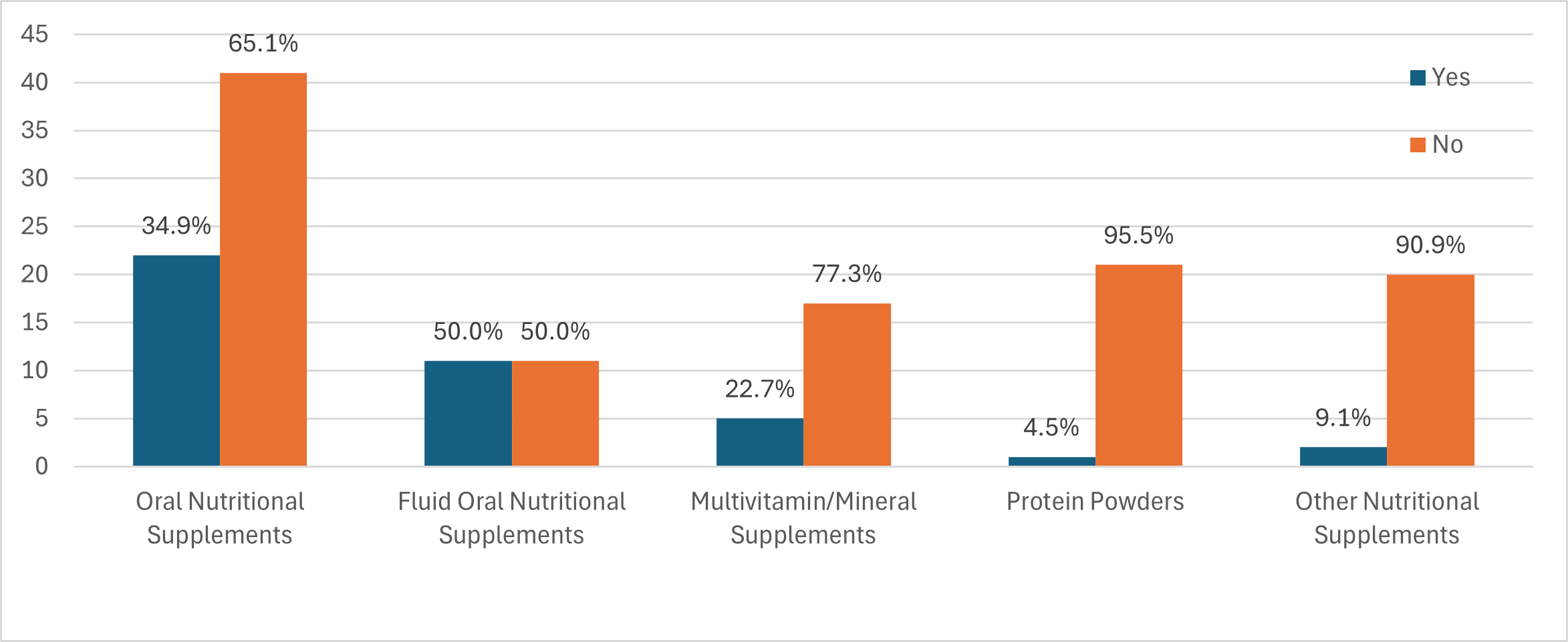

Supplement: S1 Fig — (TIFF) [file pone.0344288.s001.tiff]

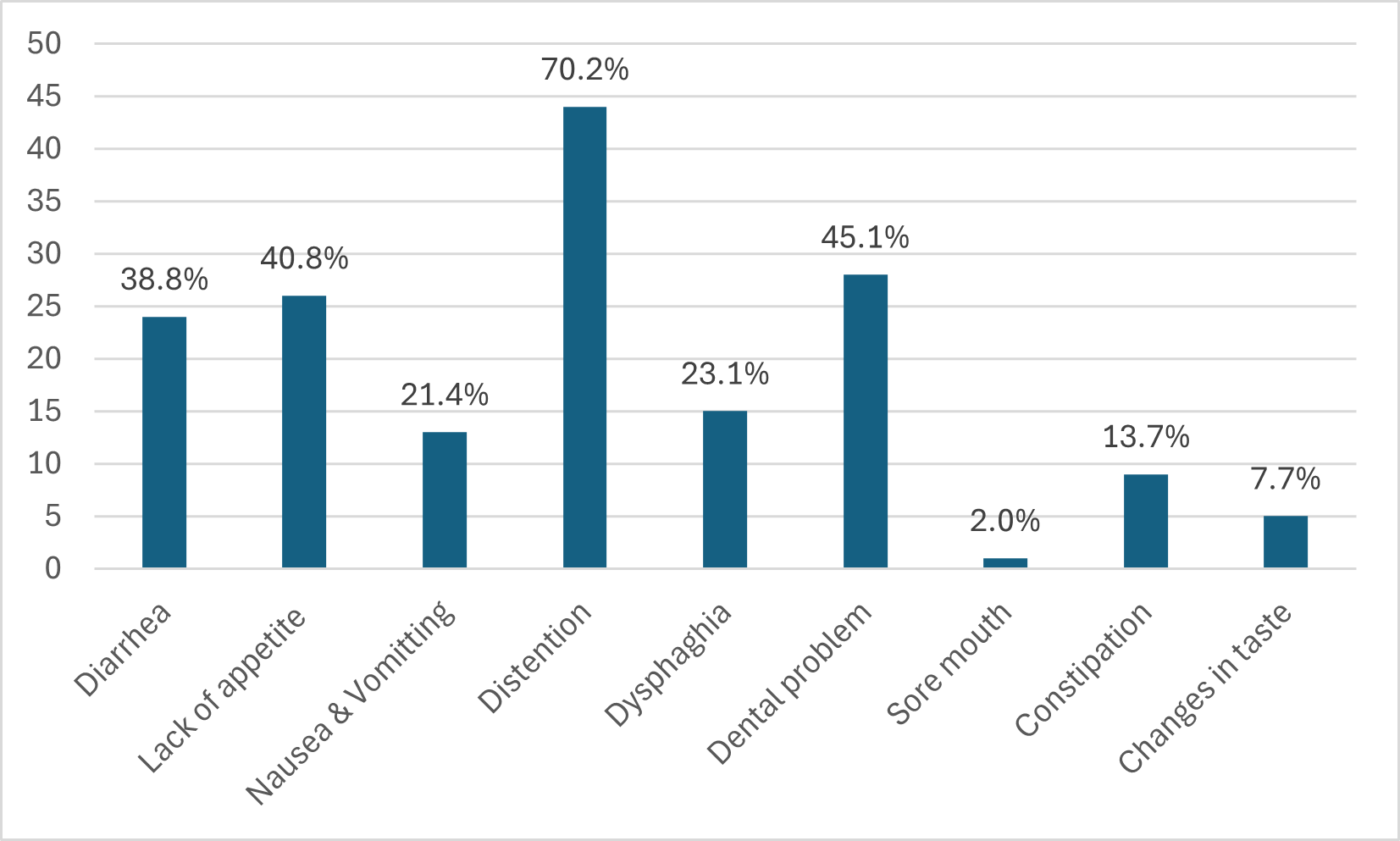

Supplement: S2 Fig — (TIFF) [file pone.0344288.s002.tiff]
