# Supplementary material for: Nutrition-related knowledge, attitudes, practice, and mental health status of people living with HIV in Dubai, United Arab Emirates: A cross-sectional descriptive study
Source: PLoS One. 2026 Mar 4;21(3):e0344288. doi: 10.1371/journal.pone.0344288 (PMC12959681; doi:10.1371/journal.pone.0344288)
Supplement: Attachment — Submitted filename: PLOS ONE KAPSTUDY HIV.docx [file pone.0344288.s003.docx]

1. References 1 and 2 reference is same
2. As of 2023, roughly 1,700 residents in the United Arab Emirates (UAE) had HIV, giving the overall population a prevalence rate of 0.02% [4]. (cite proper reference, doesn’t correspond, latest data from WHO country profile is of 2020. 2023 data with residents’ number provided in this line needs clarification. More details required in this paragraph, local outlook on the matter.
3. Line 25 - correlated with good KAP (Banwat, 2016). Not referenced, while banwat reference is present as no 27 ?
4. Line 27 – reference [7] stated about another study but not reference correct
5. Line 30 - patients often experience depression (*HIV/AIDS and Mental Health,* 2022).Citation style differing
6. The nutrition-related KAP of HIV/AIDS patients in the MENA region has not been previously

examined despite their heightened vulnerability to malnutrition. Moreover, no studies on PLHIV in the Arabian Middle East Region exist. This Statement need to thought through : as there are studies : 1. <https://journals.plos.org/plosone/article?id=10.1371/journal.pone.0288838>

2. <https://pmc.ncbi.nlm.nih.gov/articles/PMC10001308/>

3. <https://www.frontiersin.org/journals/nutrition/articles/10.3389/fnut.2024.1294233/full>

7. Line 49-50 states nutrition-related KAP and mental health status (depression and anxiety), Why was depression and anxiety taken, rationale and prevalence of the same.
8. Line 51 - in an interventional study. What interventional study ?? related??
9. Line 106 -exclusive access to the data to protect confidentiality. [23] guided all operations. Rephrase
10. Supplementary file looks like a chatgpt AI image (with background grids)
11. Demographic details – did u assess nationality ?? or region ?? or occupation ?
12. Table 3 – under knowledge *11. Which is the safest water to drink from the list?. What list ? Table 3 need refinement*
13. Line 203 - (to our knowledge), First study is enough, no requirement. If mentioning please reframe
14. Line 205- HADS scale (Zigmond & Snaith , Reference style check
15. Line 254-255 - sample corresponds to 36% of the total population of HIV patients , how can that be a strength please explain, and as it is cross sectional design – Mention the biases linked to your study, also comment on generalisability. How did you manage to overcome bias? Please mention all limitation in one subheading
16. Conclusion needs more detailing, Abstract mention prevalence of mental condition less than 20% , why quote less than 20% , Significance of the number.
17. Lastly references need through checking, some places its APA style, some references repeated, some wrongly cited.
